# Cough desensitization treatment for patients with refractory chronic cough: results of a second pilot randomized control trial

**DOI:** 10.1186/s12890-023-02423-6

**Published:** 2023-04-28

**Authors:** Laurie J. Slovarp, Jane E. Reynolds, Sophia Tolbert, Sarah Campbell, Shannon Welby, Paige Morkrid

**Affiliations:** University of Montana, School of Speech, Language, Hearing, & Occupational Sciences, Missoula, MT USA

**Keywords:** Chronic cough, Refractory chronic cough, Cough hypersensitivity, Leicester Cough Questionnaire, Cough suppression, Capsaicin, Desensitization

## Abstract

**Objective:**

The purpose of this study was to collect pilot efficacy data on a novel treatment for refractory chronic cough (RCC), which we call cough desensitization treatment (CDT).

**Design and methods:**

In this parallel cohort, sham-controlled, randomized controlled trial, 21 adults with RCC were randomly assigned to 12 sessions of either CDT (progressive doses of aerosolized capsaicin while behaviorally suppressing cough; n = 11) or a sham treatment (repeated exposure to aerosolized saline; n = 9). The Leicester Cough Questionnaire (LCQ) was the primary outcome measure. Perceived cough severity with a visual analogue scale and cough challenge testing (for measuring cough-reflex sensitivity) were secondary outcome measures. Data were analyzed with mixed effects linear regression and follow-up contrasts.

**Results:**

Results on all measures favored CDT. Excluding one sham participant, whose baseline LCQ scores were deemed unreliable, mean change in LCQ at 3-weeks post treatment was 6.35 and 2.17 in the CDT and sham groups, respectively. There was moderate to strong evidence of a greater improvement in the CDT group in total LCQ score (p = .058) and LCQ Psychological domain (p = .026) and Physical domain (p = .045) scores. Strong evidence was found for a greater reduction in urge-to-cough during CCT in the CDT group (p = .037) and marginal for a reduction in the capsaicin cough-reflex sensitivity (p = .094). There was weak evidence of a greater reduction in cough severity in the CDT group (p = .103).

**Discussion:**

Although the study is limited due to the small sample size, the data provide additional evidence supporting further research on CDT. CDT resulted in a greater change in the primary efficacy measure (LCQ) than both pharmaceutical and behavioral treatments currently found in the literature.

**Trial Registration:**

This trial (NCT05226299) was registered on Clinicaltrials.gov on 07/02/2022.

## Introduction

Refractory chronic cough (RCC)—a cough lasting more than 8 weeks that has not responded to guideline-based interventions and is not associated with smoking or lung disease—impacts up to 7 million Americans each year [1, 2]. RCC causes substantive physical and psychosocial impairments [3, 4] and places a significant economic burden on society and individuals [5–7]. Historically, RCC was thought to be a symptom of a seperate underlying chronic condition (e.g., reflux, chronic sinusitis) and the recommended treatment strategy was to systematically treat these conditions [8–10]. Research now indicates RCC is due to hypersensitivity of the cough reflex [11–13], with evidence suggesting both peripheral and central hypersensitivity [14–17]. This condition is now commonly termed *cough hypersensitivity syndrome* (CHS) [10, 11, 13, 18]. The biologic mechanisms leading to CHS are unknown, though evidence suggests an association with airway inflammation [11, 15, 19–23].

The cough reflex is a complex sensorimotor function involving interaction between peripheral and central networks [24]. An important component hypothesized to influence the human cough reflex is the urge-to-cough (UTC) which often precedes coughing. UTC is an interoceptive (i.e., internally sensed) experience driving the need or desire to cough [25] and is positively correlated with cough stimulus intensity and cough frequency [26, 27]. UTC is influenced by cognitive factors and humans can intentionally suppress cough despite UTC [28, 29], suggesting the cough reflex can be endogenously modulated through interoceptive awareness and regulation [28, 29]. Patients with RCC have a heightened cough reflex [30] and reduced ability to suppress cough relative to healthy controls [31]. This difference is associated with reduced activity in the dorsomedial prefrontal and anterior mid-cingulate cortices [32], suggesting CHS may be related to an impairment in central-mediated inhibitory networks.

Behavioral cough suppression therapy (BCST) focuses on interoceptive cues of UTC and implemention of specific cough suppression strategies in reponse to UTC [33–36]. The exact mechanism of action behind BCST is unknown, but the therapy has been shown to reduce cough frequency and severity in up to 88% of patients [22, 33–40]. Patients with RCC who have severe hypersensitivity or decreased recognition of UTC are less likely to respond to BCST.

For these reasons, we previously developed and showed preliminary evidence for a treatment approach called cough desensitization treatment (CDT), where UTC is elicited in a controlled manner in a therapeutic context to facilitate successful cough suppression. In CDT pilot 1, patients with CHS, who previously had not responded to BCST, were exposed to progressive doses of inhaled aerosolized capsaicin via a nebulizer and coached to suppress cough [41]. Following six sessions of CDT, 6/8 (75%) participants experienced a clinically meaningful improvement in cough-related quality of life as measured by the Leicester Cough Questionnaire (LCQ) [42]. In contrast, only 2/6 (33%) participants who received a sham treatment achieved a clinically meaningful improvement in LCQ.

The purpose of the current study was to expand on this work with a refined protocol given what was learned from pilot 1. Protocol modifications included: (1) a dose increase to 12 sessions, (2) additional validated outcome measures including cough-reflex sensitivity and a visual-analogue scale of cough severity, (3) revised sham treatment, and (4) expansion of inclusion criteria to include patients with RCC who had not yet received BCST. We hypothesized CDT would result in significantly improved outcomes and a reduction in cough-reflex sensitivity relative to sham treatment. The trial (NCT05226299) was registered on Clinicaltrials.gov on 07/02/2022.

## Methods/Design

### Ethics approval and participants

This randomized, parallel-group, sham-controlled trial was approved by the Food and Drug Administration (Investigational New Drug (IND) #142,148) on March 1, 2021, and the University of Montana Institutional Review Board (#188 − 18) on March 25, 2021. All methods were carried out at the University of Montana in accordance with relevant guidelines and regulations. Power analysis based on projected rate of improvement (estimated from initial pilot trial results) for each group was completed to estimate sample size. A sample size of 30 was estimated for 80% power.

Participants were recruited from speech-language pathology clinics in western Montana, a regional social media campaign, and word of mouth. Inclusion criteria included adults with a cough of at least eight weeks duration that had been treated unsuccessfully by at least one physician, normal chest x-ray and spirometry (unless diagnosed with asthma, further detail below), and no evidence of anatomical or neurological abnormality on laryngoscopy. Exclusion criteria included current smokers, individuals who were pregnant or trying to become pregnant, diagnosis of a respiratory or pulmonary condition other than asthma, positive case of COVID-19 within 14 days of enrollment or any active symptoms of COVID-19 (based on CDC guidelines), and those not currently or recently on an ACE-inhibitor or neuromodulator prescribed for cough. Individuals with asthma were allowed to enroll in the study if they were regularly followed by a pulmonologist who provided written documentation that the individual’s asthma was well controlled, and the participant had an FEV1% predicted of at least 0.60 prior to the beginning of each session. All participants signed an IRB-approved informed-consent form which included an agreement to avoid new cough treatments during the study. None of the participants in this trial were enrolled in the pilot 1 trial.

### Group allocation and blinding

The final author created a list of random numbers a-priori using a random number generator. Even numbers were assigned to the CDT group while odd numbers were assigned to the sham group. All participants were enrolled by either the first or second author. Each newly enrolled participant was allocated to the next number on the randomized participant number list. Participants were told the purpose of the study was to investigate efficacy of an inhaled substance to reduce cough hypersensitivity. The informed consent form for each group differed slightly in relation to stated treatment risks and treatment session descriptions in order to reduce risk of unblinding. Placebo participants were told only that the experimental substance was designed to reduce cough sensitivity. They were not told the experimental substance would cause an urge-to-cough (UTC) or that they would be asked to report UTC after each dose. Participants were informed the full treatment was 12 sessions across six weeks, but they would be assessed for progress after six sessions (i.e., midpoint). If no clinically meaningful progress was made on at least one measure at midpoint, they would be eligible to switch to the active treatment (if they were receiving sham) or to end treatment (if they were receiving CDT). The purpose of setting a stopping-criteria was to reduce participant burden and maximize recruitment.

Participants were blind to group assignment. It was not possible for researchers administering the treatment to be blinded because procedures for each treatment varied slightly (described below). Furthermore, there is an obvious difference in participant response when giving capsaicin versus saline. Assessors were not blinded due to budgeting and personnel constraints. To reduce risk of assessor bias, all researchers were given strict training in cough challenge testing procedures and the other outcome measures were collected via an electronic survey without involvement from the assessor other than setup assistance.

### Procedures

**Fig. 1:**
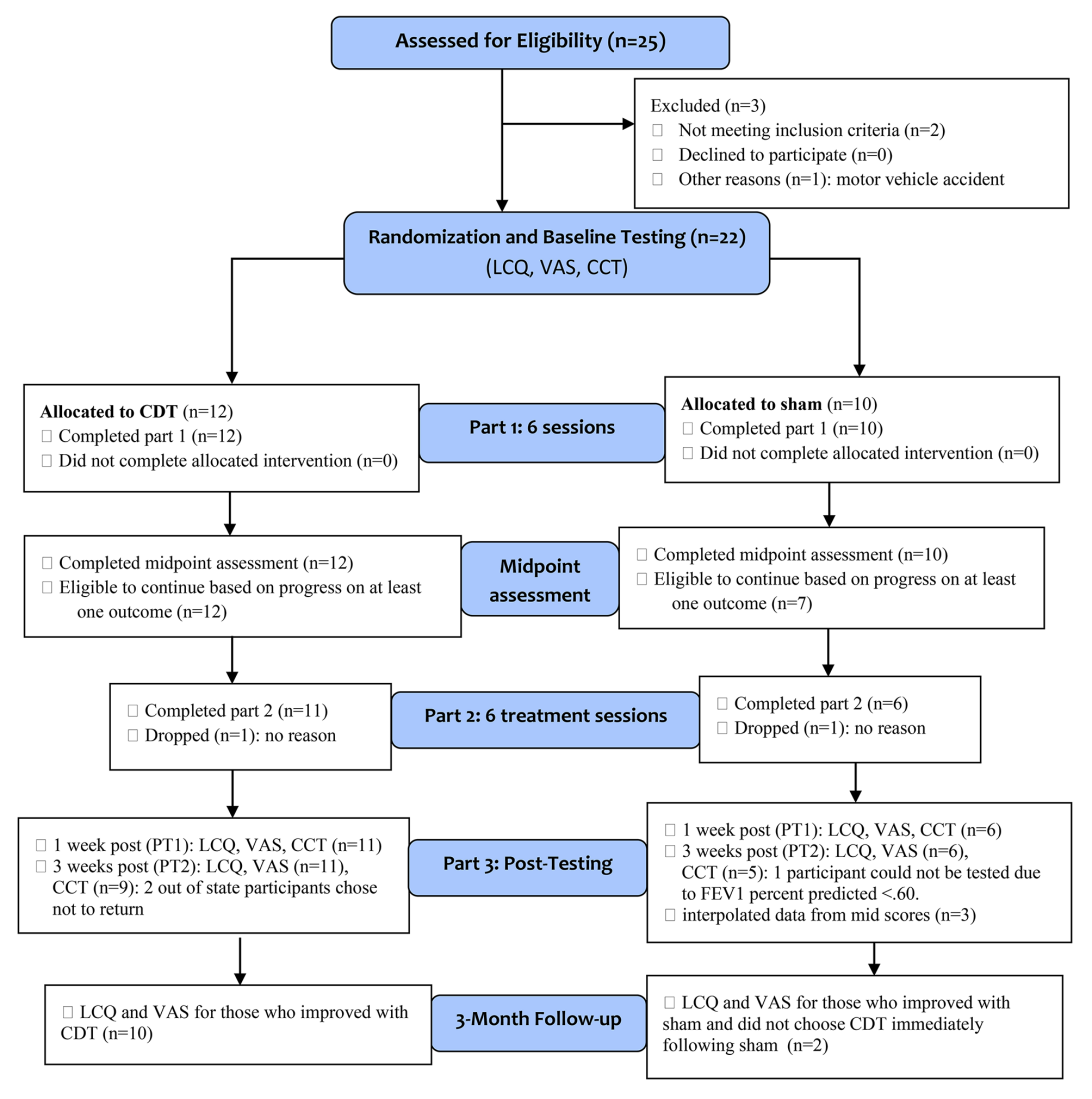
Consort Study Flow Diagram *LCQ* = Leciester Cough Questionnaire; *UTC* = urge-to-cough testing; *VAS* = visual analogue scale of cough severity; CCT = cough challenge testing; *PT1* = post-test 1; *PT2* = post-test 2

Following baseline testing, treatment sessions were delivered twice per week for three weeks, followed by a midpoint test. An increase of at least 1.30 on the LCQ [42, 43], decrease of at least 10 points on the VAS, or a C5 increase of at least two doubling doses was considered sufficient progress at midpoint to continue treatment. When treatment was discontinued at midpoint due to lack of progress, midpoint test scores were carried forward to the post-test phase for statistical analysis. All participants who completed the full treatment course were told of their group assignment after post-testing. Sham participants were then given the choice to begin CDT. Three-month follow-up data were gathered from participants who showed a clinically meaningful improvement during post-testing, unless they were in the sham condition and opted to receive CDT. A flow diagram of the study procedures is shown in Fig. 1.

### Capsaicin quality control

Pharmaceutical grade pure capsaicin was purchased from Formosa Laboratories Inc. (Formosa Laboratories, Inc. Taoyuan, Taiwan 338) and diluted in a sterile environment according to standard procedures outlined by the European Respiratory Society [44, 45]. Capsaicin was diluted with 0.95 ethanol to make 0.01 and 0.001 molar stock solutions. Stability of stock solutions was confirmed with periodic reverse-phase high-performance liquid chromatographic assay [46] before they were used and were discarded after six months of use. Stock solutions were diluted with inhalation-safe sodium chloride, using sterile procedures, for use during testing and treatment sessions. Doubling concentrations from 0.49 to 1000 μM. were made as needed within 24 h of use. Stock solutions and dilutions were protected from UV light and stored in a temperature-controlled, 4° C refrigerator.

### Outcome measures

Outcome measures were administered at baseline, 1-week post-treatment (PT1), and 3-weeks post-treatment (PT2). The Leicester Cough Questionnaire (LCQ), a validated patient-report measure of cough’s impact on quality-of-life with psychological, physical, and social domains [47], served as the primary efficacy measure. Secondary outcome measures included a visual analogue scale of cough severity (VAS) and capsaicin cough challenge testing (CCT) to measure cough-reflex sensitivity, including urge-to-cough. For the VAS, participants indicated their overall perceived cough severity by placing an “x” on a 100 mm line where the left end of the line indicated “no cough problem” and the right end of the line indicated “worst possible cough problem.” CCT is described in greater detail below. The LCQ and VAS were gathered electronically via HIPAA-compliant Qualtrics software. Two participants had traveled from out of state to participate in the study and chose not to return to the clinic for PT2 CCT. Given missing PT2 CCT data from these two participants, and the need to interpolate post-test data from the midpoint test scores for three sham participants who showed no progress at midpoint, we chose to analyze only CCT data at PT1 when looking for change in cough sensitivity. Participants who showed a clinically meaningful improvement at either PT1 or PT2, and were not sham participants who then elected to receive CDT, were asked to complete the LCQ and VAS three months following treatment.

**Cough Challenge Testing (CCT)**. CCT was used to provide two measures of cough-reflex sensitivity—C5 (i.e., concentration of capsaicin causing 5 or more coughs) and perceived urge-to-cough (UTC). Two researchers assisted during CCT sessions to ensure reliability of cough counting after each capsaicin exposure. Standardized procedures outlined by the European Respiratory Society (ERS) were followed [44, 45, 49] with a minor modification in the stopping point of the test. The QuarkSPIRO Modular Spirometry Laboratory with dosimeter (by Cosmed), was used to deliver doubling doses of aerosolized diluted capsaicin via a DeVilbiss 646 nebulizer with straw and baffle welded.^1^ The dosimeter was calibrated weekly and controlled by an inspiratory flow regulator valve. Three mL of solution were placed in the nebulizer cup before each exposure. The single inhalation method was used, with a delivery time of 0.6 s. Participants were instructed to let their body respond naturally without attempting to suppress cough. Number of coughs produced within 15 s following each exposure was manually counted by both researchers in the room. Disagreement between researchers on cough count occurred less than 5% of the time and never exceeded a difference of 1 cough. Discrepancies never questioned the C5 endpoint. Participant’s perceived maximal UTC on a modified-Borg scale from 0 (none) to 10 (very, very, very strong) was also recorded after each exposure. At least two minutes passed between each exposure to minimize tachyphylaxis. Inhalation-safe 0.9% sodium chloride (i.e., physiologic saline) was given during the first trial to ensure the participant understood the procedures and to minimize a startle effect on the initial dose. Two additional saline trials were given randomly during testing to control for anticipation effect. Following the initial saline trial, the first capsaicin dose of 0.49 μM was given. If the participant coughed less than 5 times, the next doubling dose was given and so on.

According to ERS guidelines, CCT is complete after the first dose that causes 5 or more coughs (i.e., C5) is given. However, in our prior work we’ve occasionally observed a significant unexpected drop in C5 in participants who have been tested repeatedly, leading us to question reliability of confirming C5 with only one dose. Given patients with RCC often report cough triggered randomly without apparent cause, we suspected a significant drop in C5 may be due to something other than capsaicin triggering cough. To account for this potential confound, rather than ending CCT after the first dose that caused 5 or more coughs (i.e., original C5), we continued testing with a dose of saline and then repeated the original C5 dose. If the participant coughed 5 or more times on the repeated dose, we assumed it was accurate and ended CCT. If the dose did not result in 5 or more coughs, we proceeded to the next doubling dose. Given the potential for a 15% dose-dependent tachyphylaxis for any dose preceded by the same or higher dose [50], if the participant coughed at least 5 times on the next doubling dose, original C5 was considered accurate. If the next dose did not cause at least 5 coughs, original C5 was assumed inaccurate and the test proceeded.

### Treatment procedures

Participants attended treatment sessions twice per week, with a minimum 72-hour washout period between each session (required by the FDA). The QuarkSPIRO was used to deliver either aerosolized capsaicin (CDT) or saline (sham) during single inhalations. A maximum of 12 capsaicin or saline doses were given each session. Vials containing either capsaicin or saline were stored behind a curtain, out of sight of each participant. Contrary to pilot 1, we did not encourage participants to practice cough suppression techniques, or to attempt to suppress cough, outside of treatment sessions. Both treatments were matched on number and frequency of sessions, length of sessions, and number of doses given per session.

**Cough Desensitization Treatment (CDT)**. The dose that first caused the participant to cough during baseline CCT was the first dose during session 1. After each inhalation, participants were instructed to immediately remove the nebulizer from the mouth and forcefully blow out through pursed lips or a thin straw. They were then instructed to perform cycles of relaxed throat breathing (i.e., quick nasal inhale, prolonged exhale through pursed lips or straw) until UTC subsided. Four data points were recorded after each dose: maximum UTC, discomfort (0 = no discomfort to 10 = maximum discomfort), and suppression difficulty (1 = very easy to 7 = very difficult). The goal was to gradually increase capsaicin concentration throughout each session, without coughing. The clinician considered the participant’s success with suppression and reported suppression difficulty when determining the next dose. If the participant was unable to successfully suppress, the next dose was generally reduced by a quarter or half. In the low dose range (i.e., 15.63 μM or less), if the participant did not cough and reported a suppression difficulty of 4 (moderate difficulty) or less, the next dose was doubled. If the participant did not cough and reported a suppression difficulty of 5 (somewhat difficult), the next dose was increased by half. If they did not cough but reported suppression difficulty of 6 (difficult) or higher, the dose was repeated. Guidelines were similar for concentrations greater than 15.63 μM, except that rather than increasing by half or double, they were generally increased by quarter or half. With some clinical discretion allowed, each session started at the capsaicin concentration one level below the first dose that resulted in an UTC of 3 or 4 and was successfully suppressed in the prior session. Participants were allowed to take a sip of water in between each dose.

**Sham Treatment.** Procedures were the same for the sham treatment; however, sham participants were given saline rather than capsaicin and were not instructed to do anything specific following each exposure. Nor was UTC, level of discomfort, or suppression difficulty reported following each dose. The saline was changed behind the curtain between each dose.

### Statistical analysis

Statistical analysis was performed with the statistical software R (R Core Team, 2021) with mixed models estimated using the lme4 package [51], p-values generated using lmerTest [52], and contrasts estimated using emmeans [53]. Linear or generalized linear mixed models were used with a random subject effect to account for repeated measurements (baseline, midpoint, PT1, and PT2) on each participant, with fixed effects for the repeated assessment points and group (treatment, sham) and their interaction, incorporated. First, the interaction was tested for. Then, follow-up contrasts were used to compare the differences in change from baseline to each post-test between the treatment and sham groups. Contrast p-values were adjusted for multiple testing using a family-wise Bonferroni correction. The interaction models were not corrected for the multiple response variables examined. Nonparametric tests (i.e., Mann Whitney U test and Fisher’s exact test) were completed with Statistical Package for Social Sciences (SPSS) to determine between-group differences on demographic and baseline data.

## Results

Twenty-two participants enrolled in the study. Twelve were randomized to CDT. Mean age was 64 and 50 for the CDT and sham groups, respectively. There was marginal evidence of worse total LCQ scores in the CDT group (p = .112) and strong evidence for worse VAS scores in the CDT group (p = .031). Demographics and baseline measures per group are shown in Table 1.

**Table 1:** Participant demographics and baseline outcome measures at enrollment

Characteristic	CDT	Sham	p-value
Age (SD)	64 (8)	50 (17)	0.056
Female, n (%)	8 (0.73)	6 (0.67)	1.00
Ethnicity	11 (01.00) Caucasian	8 (0.89) Caucasian 1 (0.11) Hispanic	0.450
FEV1 (SD)	2.29 (0.76)	2.79 (1.06)	0.331
FEV1% predicted (SD)	1.00 (0.31)	0.99 (0.15)	0.552
FEV1/FVC (SD)	72.88 (7.68)	73.44 (9.49)	1.00
LCQ_tot_	9.61 (2.11)	11.93 (3.66)	0.112
LCQ_Physical_	3.87 (0.95)	4.69 (1.09)	0.095
LCQ_Social_	2.98 (0.87)	3.62 (1.42)	0.152
LCQ_Psychological_	2.76 (0.64)	3.61 (1.47)	0.095
VAS	68.18 (16.61)	43.33 (25.71)	0.031
logC5	0.67 (0.57)	0.83 (0.75)	0.603
UTC at C5 dose	5.82	6.67	0.710

LCQ = Leicester Cough Questionnaire; FEV1 = forced expiratory volume in 1 s; FVC = forced vital capacity; SD = standard deviation

Every participant in the CDT group showed sufficient progress at midpoint to continue treatment. One CDT participant dropped after midpoint and declined to provide a rationale. Three participants (30%) in the sham group showed no progress at midpoint and switched to CDT. One sham participant, who met the midpoint progress threshold, dropped after midpoint without providing a rationale. The remaining participants completed the full treatment course. Midpoint scores for those who did not show progress at midpoint were carried forward to the post-test phase of the study for statistical analysis. A Consort flow diagram of the study sample is shown in Fig. 1. Results on all outcome measures are included in Table 2.

Baseline LCQ scores for one participant (whom we’ll call participant K) in the sham group were judged to be unreliable. All LCQ scores for this participant were subsequently removed. The participant chose the worst possible answer on 13 of 19 questions on the LCQ at baseline which resulted in the lowest total LCQ score (5.46) of our entire sample and nearly two standard deviations below the mean of patients with RCC, according to our prior work [35]. K’s total LCQ score improved by over 5 points post-treatment, suggesting a large improvement; yet, when answering a multiple-choice question about level of satisfaction with the treatment, K reported the lowest score (“Not at all satisfied, I’m not any better”). K’s VAS changed less than 20 points, which further suggests a less than clinically-meaningful improvement [54]. Scatterplots of satisfaction score to LCQ change for the entire sample at PT1 and PT2 showed very clear linear relationships and participant K as the only outlier. Further, Pearson product-moment correlations between satisfaction score and LCQ change with and without K in the analysis changed from.755 to 0.839 at PT1, respectively, and from 0.766 to 0.896 at PT2, respectively, providing further evidence that K’s baseline LCQ score was likely inaccurate.

**Fig. 2:**
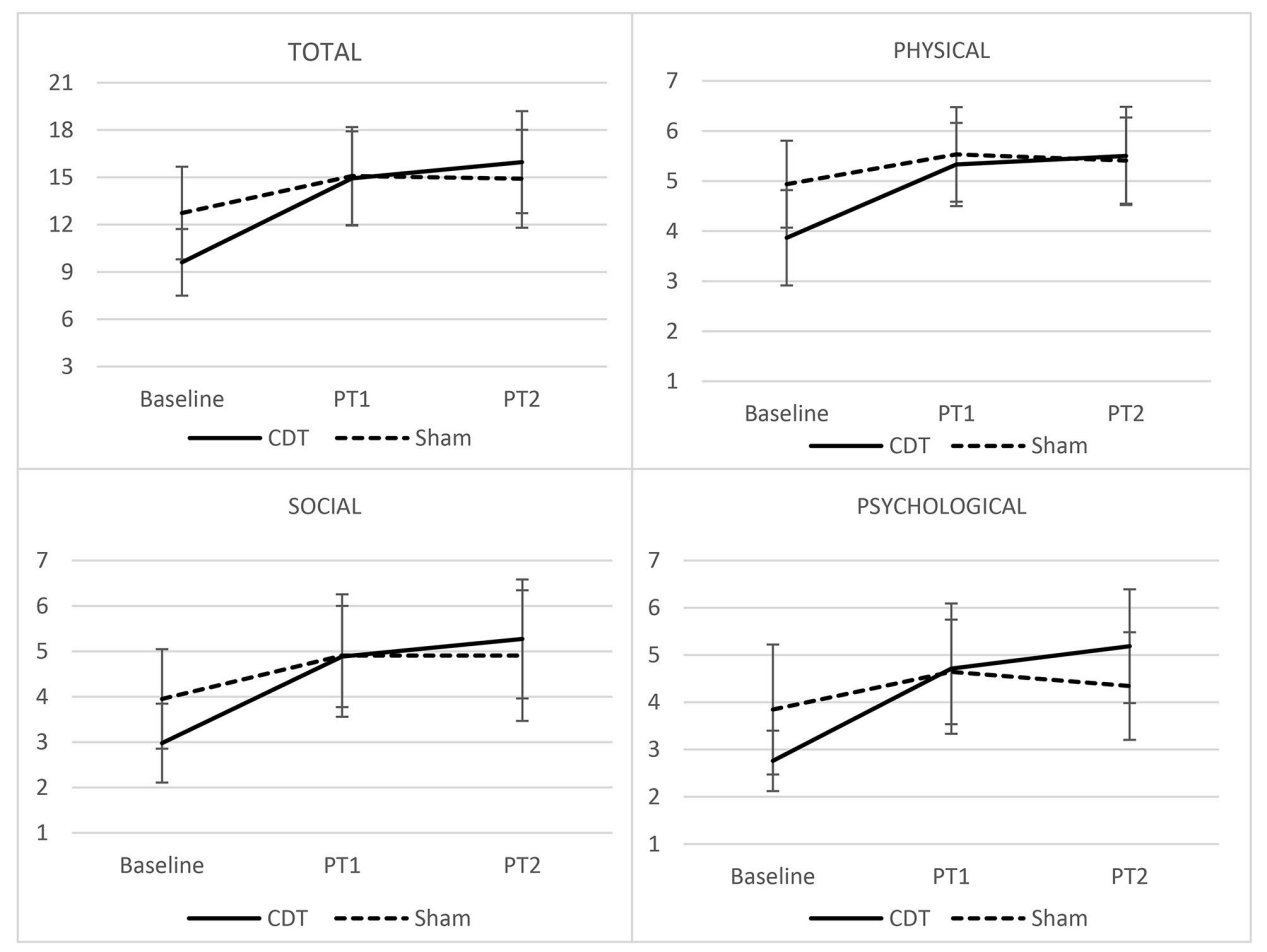
Leicester Cough Questionnaire (LCQ) total and domain scores at baseline, 1-week post-treatment and 3-weeks post-treatment per group. LCQ scores on vertical axis. Error bars indicate standard deviation

*Leicester Cough Questionnaire (LCQ)*. 91% (10/11) of CDT participants and 62.5% (5/8) of sham participants surpassed the clinically meaningful threshold change on total LCQ of 1.3 [43]. Mean change in total LCQ score was 5.32 (95% CI 3.04 to 7.60) and 2.34 (95% CI − 0.33 to 5.02) at PT1, and 6.35 (95% CI 4.07 to 8.63) and 2.17 (95% CI − 0.50 to 4.85) at PT2, in the CDT and sham groups, respectively. Mean change score in each LCQ domain were also greater in the CDT group than the sham group. There was moderate to strong evidence for a greater improvement on total LCQ score (F(3, 51) = 2.66, p = .058) and Social domain (F(2, 34) = 2.79, p = .074) in the CDT group, and strong evidence for a greater improvement on the Physical (F(2, 34)= 3.39, p = .045) and Psychological domains (F(2, 34)= 4.08, p = .026). Follow-up contrasts to estimate the change vs. baseline within each group on total LCQ revealed very strong evidence of an improvement in the CDT group at both PT1 and PT2 (p < .0001 for both) and marginal to weak evidence of an improvement in the sham group at PT1 and PT2 (p = .096 and 0.133, respectively). LCQ scores are shown in Fig. 2.

*Visual Analogue Scale (VAS) of Cough Severity.* Mean change in VAS was − 32.27 (95% CI -52.1 to -12.4) and − 5.11 (95% CI -27.0 to 16.8) at PT1 and − 33.36 (95% CI -53.2 to -13.5) and − 4.22 (95% CI -26.1 to 17.7) at PT2 in the CDT and sham groups, respectively. Mixed effects linear regression revealed weak evidence of a difference between the groups over time (F(3, 54) = 2.16, p = .103). Follow-up contrasts revealed strong evidence of an improvement in the CDT group at PT1 and PT2 (p < .001 for both) but zero evidence of a change in the sham group at PT1 or PT2 (p = 1.00 for both). VAS scores are included in Table 2.

**Fig. 3:**
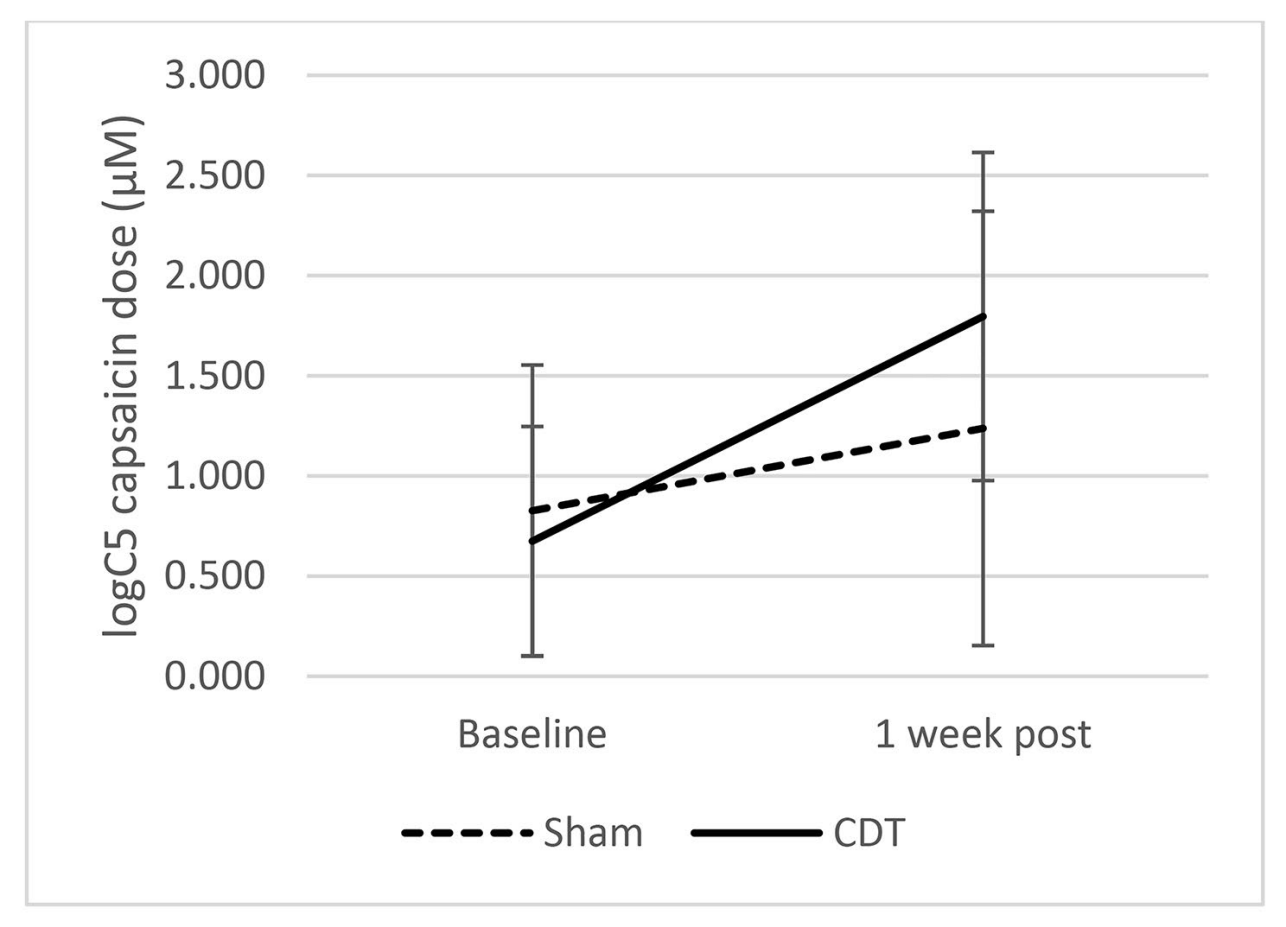
Mean logC5 score per group at baseline and 1-week post-treatment. C5 = capsaicin dose that caused five or more coughs during cough challenge testing. Error bars indicate standard deviation

*Cough challenge testing (CCT)*. Given two participants in the CDT group did not return for PT2 (because they were from out of state) and midpoint scores for three of the sham participants were carried forward due to lack of progress, we chose to only analyze CCT for PT1. Mean change in logC5 was 1.122 (95% CI 0.56 to1.68) and 0.411 (95% CI − 0.35 to 0.95) at PT1, for the CDT and sham groups, respectively. There was moderate evidence of a greater change in the CDT group over time (F(2, 35) = 2.54, p = .094). Follow-up contrasts revealed strong evidence of a reduction in cough-reflex sensitivity (i.e., larger C5 score) in the CDT group (p = .0001) and extremely weak evidence of a change in the sham group (p = .569). See Fig. 3.

**Fig. 4:**
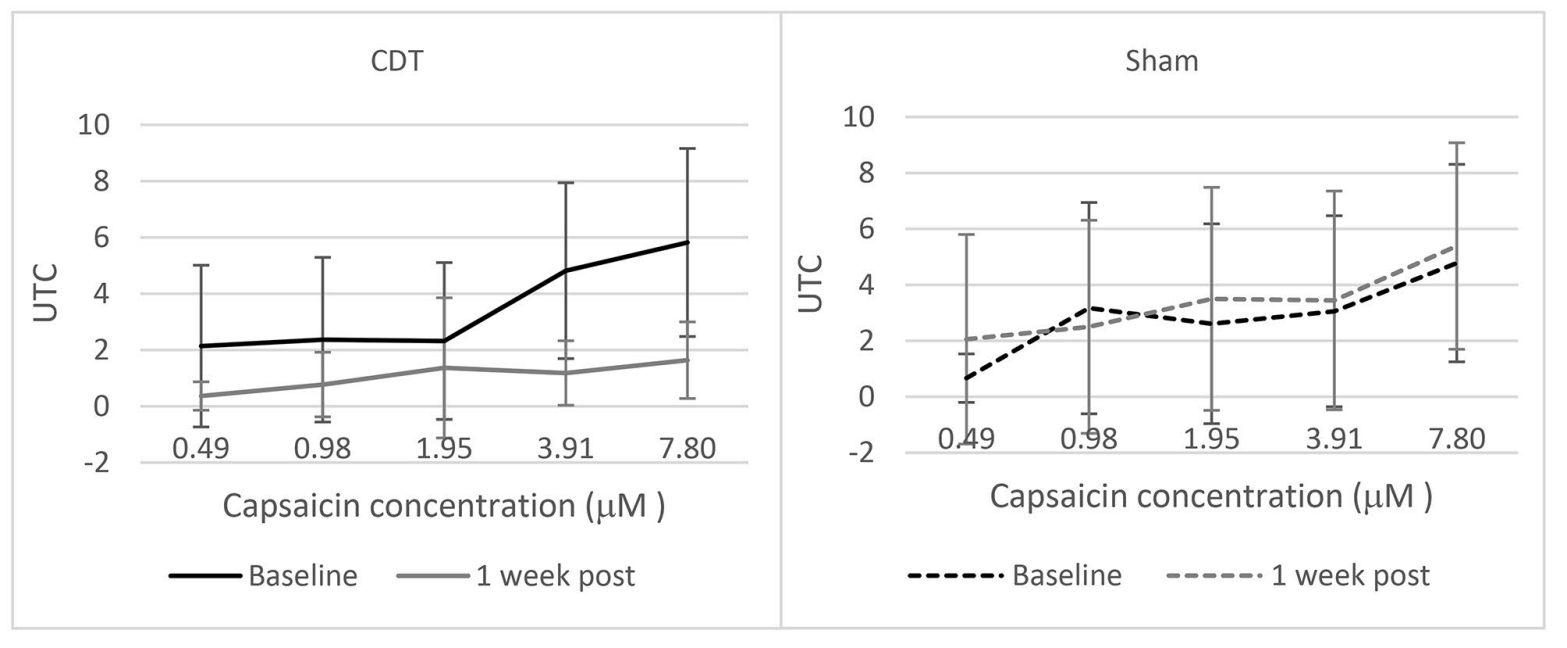
Mean change in urge-to-cough (UTC) at baseline and 1-week post-treatment (PT1) during cough challenge testing. Only concentrations 0.49 µM to 7.81 µM are shown because the majority of participants received each of these concentrations at both baseline and PT1.

*Urge-to-cough (UTC) during CCT*. Change in UTC was measured by comparing the UTC score at the C5 dose during baseline CCT to the UTC score at the same dose (baseline C5 dose) during PT1 CCT. There was strong evidence of a greater reduction in UTC in the CDT group than the sham group with mean change in UTC of -5.00 (95% CI -7.47 to -2.53) and − 1.61 (95% CI -4.34 to 1.11) for the CDT and sham groups, respectively (F(1, 18) = 3.07, p = .037). Follow-up contrasts revealed strong evidence of a change in the CDT group (p = .0002) but very weak evidence of a change in the sham group (p = .331). UTC scores for each group at baseline and PT1 up to 7.81µM are shown in Fig. 4. Concentrations larger than 7.81 µM are not included because several participants were not given concentrations beyond 7.81 µM.

**Table 2:** Change in primary and secondary outcome measures in post-treatment phase relative to baseline

	Mean Change at PT1						Mean Change at PT2							
	CDT		Sham			CDT			Sham					
	Mean	95% CI	Mean	95% CI		mean		95% CI	Mean	95% CI		N^2^_p_	F-Value	P = Value
**LCQ**_**Total**_	5.32	3.04 to 7.60	2.34	− 0.33 to 5.02		6.35		4.07 to 8.63	2.17	− 0.50 to 4.85		0.14	2.657	0.058
**LCQ**_**Phys**_	1.46	0.74 to 2.18	0.60	− 0.24 to 1.42		1.64		0.92 to 2.36	0.47	− 0.36 to 1.30		0.17	2.49	0.097
**LCQ**_**Psych**_	1.95	0.92 to 2.99	0.80	− 0.42 to 2.01		2.43		1.39 to 3.46	0.50	− 0.72 to 1.71		0.19	3.36	0.046
**LCQ**_**Soc**_	1.91	1.00 to 2.81	0.96	− 0.09 to 2.00		2.30		1.39 to 3.20	0.96	− 0.09 to 2.00		0.14	1.84	0.174
**VAS**	-32.3	-52.1 to -12.4	-5.11	-27.0 to 16.8		-33.4		-53.2 to -13.5	-4.2	-26.1 to 17.1		0.11	2.16	0.103
**logC5**	1.12	0.53 to 1.78	0.41	− 0.41 to 0.97		--		--	--	--		0.13	2.54	0.094
**UTC**	-5.00	-7.47 to -2.53	-1.61	-4.34 to 1.11		--		--	--	--		0.15	5.08	0.037

PT1 = 1-week post-treatment; PT2 = 3 weeks post-treatment; CI = confidence interval; LCQ = Leicester Cough Questionnaire; LCQ_Phys_ = LCQ Physical Domain; LCQ_Psych_ = LCQ Psychological Domain; LCQ_Soc_ = LCQ Social Domain; VAS = visual analogue scale of cough severity; logC5 = log form of dose that causes 5 or more coughs during cough challenge testing; N2p = partial eta squared effect size; UTC = urge-to-cough

*Sustainability of effect.* The LCQ was collected three months post-treatment from participants who improved by at least 1.3 on total LCQ, and who did not receive CDT following sham treatment. One CDT participant did not respond to this data request. Another reported taking several new medications since completing CDT, so their data was thrown out. This left eight CDT participants and two sham participants. Mean total LCQ at follow up for CDT participants was 15.38, which was a change of -1.81 (95% CI: -3.68 to 0.05) from PT2 but still a mean improvement of 6.16 over baseline. A Wilcoxon Signed Rank test revealed weak evidence of a difference between PT2 and follow-up (p-value = 0.180) in those treated with CDT. One sham participant changed − 4.19 LCQ points, which was 100% of gains made. The other changed − 3.84 but remained 2.66 over baseline.

*Treatment results following sham treatment*. Seven participants who completed the sham treatment elected to receive CDT. One dropped after four sessions, stating she felt bothersome throat burning after each treatment and did not think she was improving. One additional participant was dropped from the study due to inability to meet the required FEV1%-predicted threshold of 0.60 during the 5th session. This participant was diagnosed with asthma and demonstrated a variable decline in this measure as a sham participant, even missing two sessions due to not meeting the 0.60 threshold. Given this pattern, the researchers felt it prudent to refer her back to her pulmonologist. Of the five participants who completed CDT, two scored over 19 (mean change = 5.59) on total LCQ following CDT (within normal range[55]). The other three made no clinically meaningful improvement.

## Discussion

The purpose of this study was to gather additional preliminary data on a novel treatment called cough desensitization treatment (CDT), whereby patients with RCC are presented with progressive concentrations of aerosolized capsaicin while suppressing cough. While the results of our first CDT pilot RCT (pilot 1) showed a significantly greater improvement with CDT over sham (sub-threshold capsaicin) on one outcome measure, the data suggested participants would have had a greater improvement with additional treatment sessions. In pilot 2, we used a similar design but doubled the number of treatment sessions, chose aerosolized physiologic saline as the sham treatment, accepted participants who had not yet received BCST, and eliminated the component of suppressing cough outside of treatment sessions.

As hypothesized, a greater number of CDT sessions resulted in a greater improvement—6.35 vs. 3.20 change in total LCQ. The sham group also achieved a greater improvement in pilot 2—2.73 vs. 1.75. The result in the sham group is interesting given that change in total LCQ with a sham or placebo treatment in prior RCC randomized control trials has not been more than 1.2 (range − 0.8 to 1.2) in pharmaceutical studies [56–59] and 1.66 in the one BCST study that included a placebo and the LCQ [34]. We chose saline as the sham treatment in the current study because we suspected the improvement in the sham group in pilot 1 may have been due to subthreshold diluted capsaicin. However, given capsaicin dilutions are diluted with saline, it may be that nebulized saline provided a therapeutic benefit to sham participants in both studies. This would not be entirely surprising given the known benefit of nebulized saline on laryngeal hydration [60, 61], and evidence supporting BCST, which includes emphasis on hydration [33, 34, 40, 62]. Of course, it’s also possible the intensity of the treatment (i.e., 12 sessions) resulted in a greater than expected placebo effect. Additional research is needed to determine if nebulized saline is in fact therapeutic for patients with RCC.

The therapeutic outcome of 12 sessions of CDT, as measured by the LCQ, surpasses pharmaceutical RCC treatments in the literature. As can be seen in Table 3, one can expect a change of about 2.5 LCQ points for neuromodulators [56, 57], 3.2 points for extended-release low-dose morphine [58], and 3.0 to 3.5 points for P2X3 antagonists [59, 63]. Given participants in the current study did not receive BCST prior to CDT, it could be argued the participants simply learned how to suppress their cough during CDT and then implemented those strategies outside of treatment, essentially replicating BCST. Fortunately, there are eight BCST studies that have included the LCQ to compare to. The mean change in total LCQ across these studies is 4.03 points (95% CI: 2.84 to 5.18; see Table 3) [34, 35, 37, 40, 64–67]. Given the minimum clinically-meaningful change in LCQ is 1.3 to 2.3 points in patients with refractory chronic cough [43], and CDT resulted in a change in LCQ 2.32 points greater than the BCST mean, and 1.17 points greater than the upper 95% confidence interval, our data suggests CDT is superior to BCST. Further research comparing BCST to CDT, with a larger sample size, is certainly needed to confirm this finding.

Vertigan et al. (2016) found combining BCST with pregabalin resulted in a mean change in total LCQ of 6.6 points, which was significantly greater than BCST plus placebo. However, two weeks after discontinuing pregabalin, LCQ change dropped in the BCST + pregabalin group and was no longer different than those treated with BCST + placebo. Additionally, patients taking pregabalin reported cognitive changes (30%), dizziness (45%), and weight gain (25%). In contrast, only one adverse effect (temporary throat irritation) was reported with CDT.

**Table 3:** Mean change in total LCQ score across RCC clinical trials

Treatment	Author	Study Design	LCQ change
BCST	Ryan et al., 2009^38^	Prospective case series	7.1
	Ryan et al., 2010^39^	Prospective case series	3.4
	Patel et al., 2011^66^	Prospective case series	2.7
	Vertigan et al., 2016^67^	Parallel RCT	3.3
	Chamberlain et al., 2016^35^	Parallel RCT	3.4
	Wright et al., 2021^68^	Prospective case series	4.4
	Slovarp et al., 2021^36^	Prospective case series	4.6
	Kapela et al., 2020^69^	Prospective RCT	3.2
Neuromodulators	Ryan et al. 2012^59^	Parallel RCT	2.5
	Bowen et al. 2018^58^	Prospective cohort	2.5
BCST + Pregabalin	Vertigan et al. 2016^67^	Parallel RCT	6.6
Opiate	Morice et al. 2007^60^	Crossover RCT	3.2
P2X3 antagonist	Smith et al. 2020^61^	Crossover RCT	3.6
	Morice et al. 2021^65^	Crossover RCT	2.3

Superscript number indicates citation reference number

Given CDT was designed to reduce cough-reflex sensitivity, we expected to find stronger evidence of a between-group difference in capsaicin cough challenge testing (CCT). The strength of the evidence may have been limited due to reduced sensitivity of CCT procedures, which were validated and tested for reliability on healthy individuals rather than patients with RCC, a concern also pointed out by Hilton et al. [50]. Current ERS guidelines for single-inhalation CCT is to provide doubling doses of diluted capsaicin until finding the first dose that causes five or more coughs (i.e., C5) [45]. However, given our observation of occasional unexpectedly low C5 dose in patients we have tested multiple times, we chose to take steps to improve confidence in finding the accurate C5 endpoint. Rather than concluding CCT after finding the first dose that caused five or more coughs, which we’ll call “original C5”, we chose to provide a saline dose and then repeat the original C5 dose. If the repeated dose caused five or more coughs, we felt confident in assigning C5 to the original C5 dose; however, if the repeated dose did not cause five or more coughs, we proceeded to the next doubling dose and so on. (See Methods for exact procedures.) Using this modified approach, assigned C5 was different than original C5 a total of 15 times across 74 (20%) cough challenge tests. Given reproducibility studies [68, 69] indicate acceptable reproducibility within two doubling doses of the original test result, we looked at how many doubling doses assigned C5 was from original C5. This accounted for seven of the 15 instances. Of the remaining eight, two were three doses higher than the original C5. The remaining six were four or more doses higher than original C5. In the most extreme case, a participant coughed six times on .98μM, did not cough again until given 62.5mM, and did not reach the assigned C5 until 500μM – nine doubling doses from the original C5.

CCT reliability studies were conducted on healthy individuals, which may not accurately translate to disease populations. Patients with RCC commonly cough without a specific triggering event, making it quite feasible that a patient with RCC will exhibit a cough at some point during CCT that has nothing to do with capsaicin exposure. If this happens within 15 s following a capsaicin exposure during CCT, it could easily result in a false positive test. We would not expect to see this pattern in healthy individuals who are not likely to experience unexpected cough triggering.

Hilton et al. also questioned current ERS CCT guidelines [50]. They completed an elegant capsaicin dose-response study using pharmacodynamic modeling that included healthy volunteers, patients with chronic cough, and patients with asthma. Participants were given doubling doses to the highest tolerated dose. Maximal cough response (i.e., E_max_) better discriminated between health and disease than either C2 or C5. E_max_ resulted in much less between-subject variability than C2 or C5. In contrast, our results emphasize the potential problem of within-subject variability. Collectively, these two studies suggest additional research is needed contrasting validity, feasibility, reproducibility, and repeatability of cough sensitivity testing via a true dose-response testing paradigm (i.e., E_max_ and ED50 endpoints) versus the C2 or C5 endpoints in patients with RCC.

Although this study was not designed to look specifically at changes in peripheral and/or central nervous system mechanisms of the cough reflex, the results do provide clues towards understanding how both CDT and BCST may work. We found the biggest difference between CDT and sham groups when measuring UTC during CCT. From this we can tentatively infer that CDT may have resulted in a change in interoceptive processing of vagal afferent information, or that overtly attending to UTC (i.e., strengthening interoception), while attempting to change the desired motor output response (i.e., avoid coughing), increased activation of the inhibitory motor network that has been shown by Ando and colleagues to be decreased in patients with RCC [32]. If this is the case, it is likely that BCST, which similarly focuses on interoception and cough suppression, works on the same mechanism. However, given CDT involves repeated peripheral stimulation with capsaicin, which has been shown to reduce neuropathic pain and non-allergic rhinitis via desensitization, it’s also reasonable to hypothesize that CDT may result in both central and peripheral changes. This same principle has been investigated in the form of a capsaicin pill with some promise [70]; however, given rapid metabolization of capsaicin, desensitization is unlikely to be the mechanism at work in this case [71]. Investigation with fMRI and transcriptomic analysis of airway mucosal biopsies pre and post CDT and BCST would help elucidate physiologic mechanisms underlying these treatments.

In addition to the limitation related to objective cough sensory testing, as described above, there are several other limitations that warrant discussion. The first and foremost is the small sample size, which certainly reduces the power of the study. While we had hoped to obtain a larger sample size, the study was funded by a one-year grant (P20GM103474) and additional funding was not available to the authors to continue the study. Rather than waiting for additional funding to resume the study, it seemed prudent to look at the data to determine if sufficient preliminary data had been obtained to justify a large-scale, multiple-site and multiple-year clinical trial. We are confident our data does provide such justification.

Additional limitations include interpolation of midpoint test scores as PT1 and PT2 scores in three sham participants who did not show progress at midpoint. It is unknown if these participants would have shown progress with another six sessions. To minimize participant burden and maximize recruitment, we felt it important to provide a reasonable stopping point for all participants if they were not improving. There were zero CDT participants who showed no progress at midpoint, which suggests it was an appropriate time to assess for progress.

### Future directions

Many unknowns remain about CDT. It is unclear how long the CDT treatment effect lasts, or if titration of the treatment may result in greater sustainability of effect rather than an abrupt discontinuation of the treatment. The optimal dosing schedule for CDT is also unknown. Delivering the treatment more often (e.g., 3–4 times/week) may be more efficient; however, given lack of studies on safety of repeated exposure to aerosolized capsaicin, the FDA recommended a washout period of at least 72 h between treatment sessions. There is also much to be learned about what patients are the best candidates for CDT and how to feasibly implement CDT in the clinic in a cost-effective and accessible manner. Additional studies with larger sample sizes are needed. Matching participants on cough severity at baseline would also be beneficial.

## Conclusions

This study provides further preliminary data supporting a novel treatment for patients with RCC—cough desensitization treatment (CDT). CDT resulted in greater improvement in cough-related quality of life than both pharmaceutical and behavioral treatments reported in the literature, providing strong evidence to support a phase 2 clinical trial. If clinical trial results continue as seen in this preliminary work, CDT could positively impact millions of individuals worldwide who suffer from the debilitating effects of RCC.

## Data Availability

The datasets used and/or analyzed during the current study are available from the corresponding author on reasonable request.

## Footnotes

1 ERS guidelines recommend the KoKo Digidoser; however, the KoKo is no longer manufactured or supported. The QuarkSPIRO equipment has the exact same specifications as the KoKo Digidoser.
